# Enhanced Frying Efficiency at Low Temperatures Utilizing a Novel Planetary Fryer

**DOI:** 10.3390/foods13121896

**Published:** 2024-06-17

**Authors:** John S. Lioumbas, Despoina Anastasopoulou, Maria Vlachou, Margaritis Kostoglou, Theodoros Karapantsios

**Affiliations:** School of Chemistry, Aristotle University of Thessaloniki, 54 124 Thessaloniki, Greece; ydanastaso@gmail.com (D.A.); marvlach@gmail.com (M.V.); kostoglu@chem.auth.gr (M.K.); karapant@chem.auth.gr (T.K.)

**Keywords:** planetary motion frying, low-temperature frying, heat and mass transfer optimization

## Abstract

This study aims to optimize the frying process of natural porous materials (like potatoes) by enhancing heat and mass transfer phenomena through significant horizontal acceleration values following a spatially periodic pattern that alternates the intensity of inertia forces uniformly across the frying vessel. The generated horizontal inertial forces act complementary to the normal vertical buoyancy force for the creation of agitating convective currents in the oil and for vapor bubbles’ departure from the surface of frying objects. The use of an innovative frying device, employing simultaneous rotation around two vertical axes at a different speed in a so-called planetary type of motion, serves to facilitate this production of horizontal acceleration values that allows intensifying the performance of frying. The present investigation examines the impact of rotational speed, along with oil temperature and frying duration, on the water loss and sensory evaluation of fried items. The potato-to-oil ratios typically found in industrial frying operations are employed. The intended outcome is a more energy-efficient frying process, reduced cooking times, and a healthier product due to lower frying temperatures and the consequent decreased formation of harmful compounds. This approach carries substantial implications for food processing, potentially enhancing productivity while limiting operational costs.

## 1. Introduction

The complex process of deep-fat frying, commonly known as frying, involves numerous physical and physicochemical transformations [1]. This culinary process includes phenomena related to heat and moisture transfer in natural porous material such as potatoes [2], which become even more complicated as the crust grows [3]. At the same time, the transformation of water into vapor results in the formation and expulsion of vapor bubbles from the food surface, further escalating the complexity of the process [4]. The dynamic behavior of these vapor bubbles plays a critical role, as it directly influences the quality and uniformity of the end product [3]. Specifically, the buoyancy forces have been projected to significantly impact the bubble formation, growth, and detachment from the potato surface [5].

Therefore, understanding and managing the dynamics of the vapor bubbles during the frying process becomes a pivotal aspect in optimizing this culinary procedure [6]. A well-regulated heat transfer achieves equilibrium between exterior crispiness and interior moisture retention, contributing to the rich textures and flavors found in fried foods [2]. Moreover, advancement in heat transfer efficiency could allow frying at lower temperatures and shorter cooking times, leading to reduced waiting periods for consumers and heightened productivity in commercial kitchens, a potential practice for boosting the frying process [1]. From the perspective of energy conservation and cost efficiency, the practice of frying at reduced temperatures diminishes the energy demand required to maintain elevated oil temperatures, resulting in substantial energy savings [7] and decreased operational costs in commercial environments [8]. Additionally, lower frying temperatures correlate with a diminished formation of harmful compounds such as acrylamide [9,10], producing a healthier consumable product. This advancement would constitute a significant leap forward in the ongoing initiatives to diminish the health risks associated with acrylamide consumption—a primary objective for consumers, food manufacturers, and regulatory authorities alike [11].

Acknowledging the potential of the above research topics, our laboratory, supported by the European Space Agency (ESA), spent the last decade exploring the heat and mass transfer phenomena during frying, specifically scrutinizing the feasibility of producing fried foods in zero-gravity conditions. Our first studies underscored the critical influence of potato orientation on the propagation of the evaporation front and the resultant crust thickness during deep-fat frying [12,13,14]. Subsequent research showed the effect of buoyancy on boiling within porous media under hypergravity conditions, illuminating the crucial role that gravitational acceleration plays in the boiling process [15]. In addition, a study focusing on the dynamics of bubbles and substrate thermalization during boiling in a water-saturated porous matrix revealed a considerable impact of the bubble dynamics on the heat and mass transfer phenomena during frying [16]. A thermal analysis conducted during the pre-boiling stage of frying experiments stressed the dependency of heat transfer coefficients on various variables, including oil temperature, fryer geometry, the orientation of the frying surface, and gravity levels [17]. Our most recent study, examining the viability of frying in space, suggested the feasibility of accomplishing frying even under diminished gravitational acceleration [5].

Researchers have studied various aspects of frying process optimization. Vacuum frying reduces oil absorption and achieves healthier fried foods [18]. Pulsed Electric Field (PEF) treatment pre-frying can enhance heat transfer and decrease oil uptake [19]. Ultrasonic frying, which uses ultrasonic waves, can also minimize frying time and oil uptake [20]. Edible coatings before frying can reduce oil absorption and improve texture [21]. Moreira [22] and Moreira and Barrufet [23] focused on mass transfer, particularly of moisture and fat content, in deep-fat frying. Others showed that infrared heating as pre-treatment can partially cook food and reduce oil uptake [24].

The oil type affects frying efficiency by altering heat and mass transfer [1]. In this study, we used extra virgin olive oil (EVOO) as a frying medium because of its numerous health benefits, oxidative stability, and sensory qualities. EVOO is a fundamental component of the Mediterranean diet, renowned for its high content of monounsaturated fats and antioxidants, which provide anti-inflammatory and cardiovascular health benefits. Recent studies [25] have shown that EVOO maintains its stability and beneficial properties even after multiple frying cycles. Furthermore, EVOO produces fewer harmful oxidation products and retains a significant portion of its antioxidants during the frying process, enhancing the nutritional profile of the fried food [26,27]. Our collaboration with the European Space Agency (ESA) informed our choice of olive oil due to its recommendation for astronauts’ diets on missions to the Moon and Mars, given its nutritional quality and oxidative stability, making it ideal for specialized applications like space travel. Moreover, by using EVOO, which presents a worst-case scenario due to its higher viscosity and lower smoke point, we thoroughly assessed the performance and robustness of our proposed planetary fryer design. This approach ensures that if the fryer demonstrates effective functionality with EVOO, it will likely perform exceptionally well with oils possessing more favorable frying properties.

The current research diverges from previous studies on the way of intensification of the frying process, as it primarily focuses on enhancing the heat transfer through the manipulation of buoyancy forces during frying, which probably results in a bubble dynamics modification on the potato surface. This approach represents a novel perspective in the field, aiming to optimize the frying process by adjusting these key physical parameters. Experiments will be conducted using an innovative frying device [28], designed with an emphasis on optimizing bubble dynamics to augment the overall efficiency of frying. This innovative device employs a simultaneous dual rotation strategy, where the frying container rotates around its axis and the central axis of the device, mirroring the forces associated with planetary mills to enhance heat and mass transfer during the frying process. At the same time, the bidirectional rotation induces a complex movement pattern within the frying container, augmenting even heat distribution and mass transfer, thereby facilitating uniform frying. Crucial factors such as oil temperature, frying duration, and rotational speed will be examined for their influence on frying efficiency. The objective is to fine-tune these parameters to yield high-quality frying outcomes, all while conserving oil quantity and reducing cooking time.

## 2. Materials and Methods

### 2.1. Description of the Planetary Fryer

**Figure 1:**
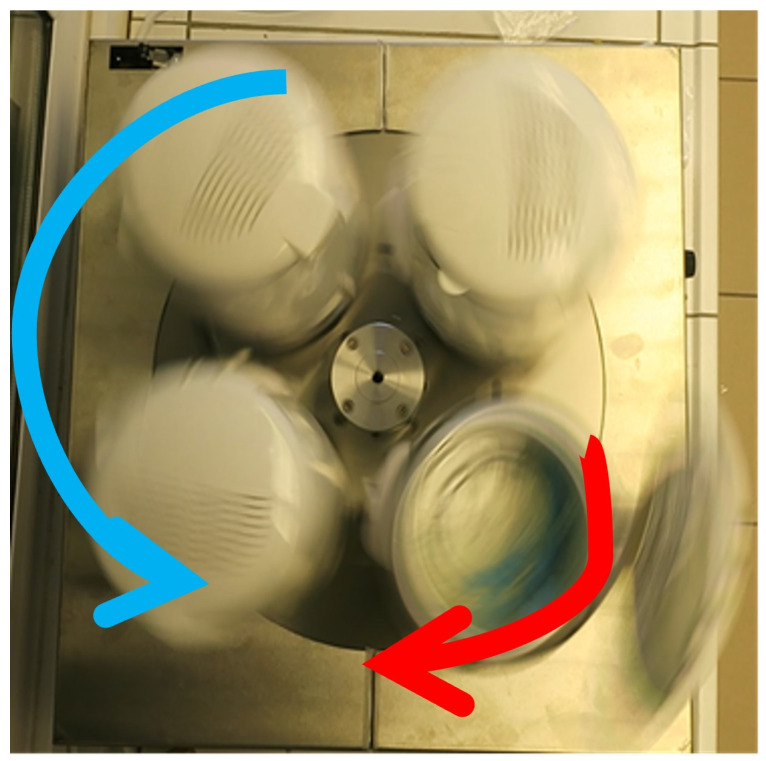
Depiction of the planetary fryer during operation. Red arrow indicates the rotational motion around the center of symmetry of the frying vessel. Blue arrow indicates the circular motion around a point outside the frying vessel (related video can be found in Supplementary File S1).

The concept of modulating acceleration forces during the frying process is inspired by the operational principles of planetary mills [24]. In the experimental setup used for this study, a distinct form of planetary motion is implemented. This involves four identical fryers (840 w, 0.9 L, Russell Hobbs Compact Deep Fryer 18238, Failsworth, Greater Manchester, UK) arrayed in a cross-shaped configuration, each rotating around a central axis situated at the core of the arrangement (Figure 1). The desired rotation speed was set at a range from 0 rpm to 250 rpm, depending on the experiment phase. Moreover, each fryer features its rotation around its axis. This complex rotational movement is enabled by a carefully designed gear system, engineered in such a way that it produces the necessary relative rotation between the two rotating components. Notably, the rotation of each individual fryer is calibrated to be exactly a 1/4 fraction of the rotation of the main central axis (a video showing the planetary motion of the fryer is provided in Supplementary File S1). This arrangement allows for an increase in effective acceleration in each fryer without additional energy demand on the central axis. Consequently, the design efficiently optimizes acceleration and rotational energy usage within the system.

The simultaneous rotation of the frying basket around its axis and the central axis of the device prompts an intricate synergy to generate a very complicated flow field in the oil. In the absence of rotation, the main heat transfer mechanisms include the natural convection of oil motion and the forced convection generated by bubble motion. Both these mechanisms are dominated by the existence of an acceleration field (gravitational at the absence of rotation). This simultaneous rotation enhances the acceleration field, increasing the efficiency of the heat transfer mechanisms. Both centrifugal and Corriolis forces are applied to the oil and to the potatoes as they are moving in the fryer. Details concerning the trajectories calculations are presented in Supplementary File S2.

### 2.2. Description of the Frying Process

Fresh potato tubers (Agria variety, all from the same producer, geographical region, and harvesting period) were stored and conditioned under regulated temperature and relative humidity [28], and then peeled and cut into 1 cm × 1.2 cm × 3 cm pieces using a spatula and thickness gauge. Extra virgin olive oil with an acidity of less than 0.8% from ALTIS has been used as the frying medium. One frying load, F, was employed (i.e., F = 1/7 kg_potatoes_/L_oil_), which resembles the potato-to-oil ratios used on an industrial scale (i.e., 55 g of potatoes at 385 mL of oil [12]). Then, the desired amount of oil (385 mL) was added to the fryer. A 0.1 g precision balance (Denner Instrument) was used to record the weight of the potato before and after frying. Each sequence of experiments was performed three times, with the figures presenting the average values. The standard deviation across these experiments was consistently less than 5%, indicating high reliability and precision in our measurements. To enhance the transparency and interpretability of our results, a comprehensive statistical analysis was conducted, and error bars were included in the graphical representations to visually indicate the variability and reliability of the data.

**Figure 2:**
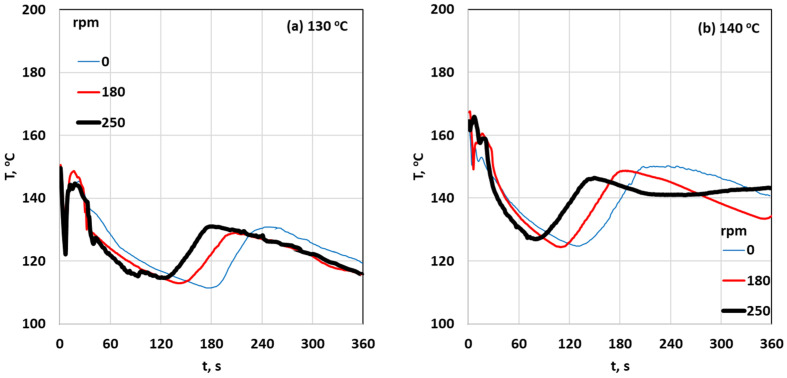
Typical olive oil temperature profiles during a standard frying process at several rotational speeds (0, 180, and 250 rpm) at (**a**) initial oil temperature of 130 °C and (**b**) initial oil temperature of 140 °C.

A K-thermocouple was connected to the temperature recorder and the fryer temperature was set to the required point, either 130 °C or 140 °C. That thermocouple was then utilized to regulate the temperature through the fryer on-off controller. Once the oil temperature reached the set point, the prepared potatoes were added to the fryer. The required frying time, either 4 or 6 min, was measured. After frying, the difference in mass was measured to understand the impact of frying on the potato weight. After frying and measurement, photographs of the fried potatoes and their cross-sections were taken for further analysis (Canon PowerShot G9 X Mark II; analysis of images: 5472 × 3648 pixels). Figure 2 presents typical olive oil temperature profiles, T(t), obtained at various frying conditions (i.e., for two initial oil temperatures (i.e., T_oil_ = 130 and 140 °C) without rotation (0 rpm) and two indicative rotational speeds (i.e., 180, 250 rpm) during a standard frying process). Upon immersion of the potatoes in hot oil, a noticeable decrease in oil temperature from its initial value is observed. This decrease is significantly more pronounced at elevated initial oil temperatures. Additionally, as the rotation speed increases, the minimum temperature value is reached at progressively earlier times, which provides an initial indication that the intensity of heat and mass transfer phenomena grows with the strength of the rotational field.

The calculation of the total heat flux is based on calculating the energy required for water to evaporate during the process. This can be determined by measuring the mass loss of water during the whole duration of frying (i.e., 4 or 6 min) and converting it into a time-based rate. By incorporating the latent heat of the vaporization of water (2257 kJ/kg), the energy expended on water evaporation per the total drying duration can be calculated. Subsequently, the heat flux is computed by dividing this energy by the surface area of the potatoes (calculated by the known dimensions of the potato sticks and their number, as equal to 16.4 cm^2^). This method provides a valuable estimation of the heat flux involved in frying processes, offering insights into the thermal energy dynamics during the frying process.

The flavor profile of the potatoes was examined scientifically by deploying an expert panel, adopting a methodological approach akin to the one proposed by Tomlins [28]. This panel, consisting of students, researchers, and professors, conducted a taste evaluation and completed questionnaires to validate the results. The sensory tests were complemented by measurements of the moisture content of the final product and monitoring of moisture levels at various stages during frying. Additionally, qualitative assessments of color, overall visual appearance, uniformity of frying across the entire potato surface, and the thickness of the crust were conducted. These combined assessments ensured a comprehensive and scientifically rigorous evaluation of the planetary fryer’s performance.

## 3. Results

### 3.1. Initial Observations Confirmed through Sensory Validation

**Figure 3:**
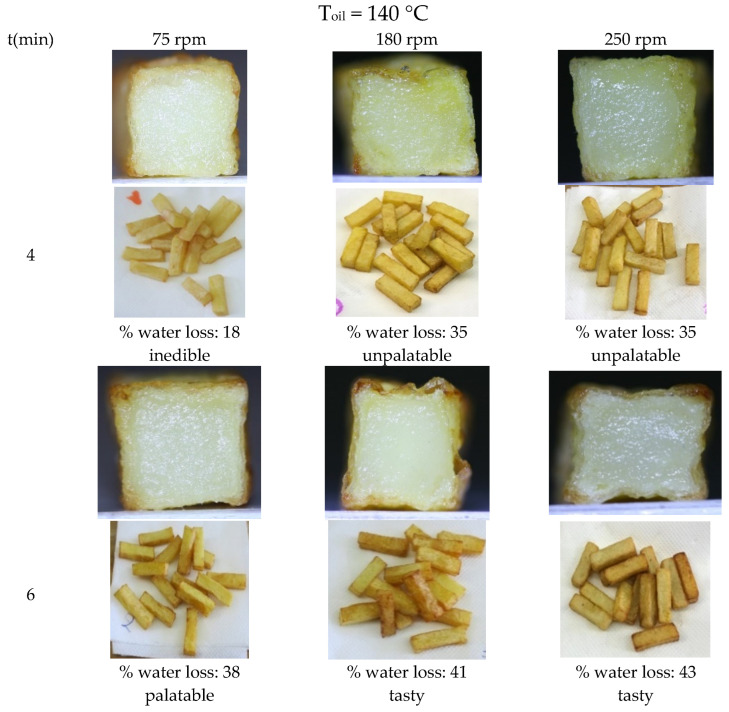
Impact of frying duration and rotational speed with water loss and resultant quality of fried potato products. The figure illustrates the influence of varying rotational speeds (75, 180, 250 rpm) and frying durations (4 and 6 min) at a constant oil temperature of 140 °C. The resulting percentage of water loss is mapped against a qualitative scale of product acceptance, from ‘inedible’ to ‘tasty’. Cross-sectional images at each condition depict the corresponding crust development.

Figure 3 presents the relationship between frying duration, rotational speed, water loss, and the resultant culinary quality of fried potato products. The results are presented from typical frying experiments conducted at a constant oil temperature of 140 °C, with varying rotational speeds (75, 180, and 250 rpm) and frying durations (4 and 6 min) as the key variables. A correlation is observed between the % water loss and the resultant quality of the fried potatoes, categorized qualitatively at four scales of acceptance varying from ‘inedible’ to ‘tasty’. For example, after a 4 min frying duration at the lowest rotational speed of 75 rpm, the potato product loses only 18% of its water content. This minimal water loss translates to an ‘inedible’ product, evidenced by the absence of a crust, a crucial characteristic of well-fried potatoes, as shown in the corresponding cross-sectional image.

Even when the rotational speed is increased to 180 and 250 rpm within the same 4 min frying duration, the water loss increases to 35%, leading to a product only marginally better, labeled as ‘unpalatable’. The respective cross-sectional images which present the crust development show a preliminary crust formation; however, the crust is not sufficiently developed to significantly enhance the product palatability. Interestingly, an increase in frying duration to 6 min at a rotational speed of 75 rpm has a significant impact on the culinary quality of the product. A water loss of 38% is attained, leading to a noticeable, albeit softer, crust development that renders the potato product ‘palatable’. The corresponding image substantiates this quality upgrade, showcasing a marked difference in crust development compared to the previous conditions. A noteworthy finding is that the product’s palatability is elevated further, achieving a ‘tasty’ rating, when the water loss surpasses the 38% threshold. This enhancement occurs under the higher rotational speeds of 180 and 250 RPM, combined with an extended 6 min frying duration, resulting in water losses of 41% and 43%, respectively. This elevated water loss is accompanied by the formation of a well-defined, robust crust, distinctly separated from the potato’s moist core.

### 3.2. Effect of the Rotational Field on % Water Weight Loss

**Figure 4:**
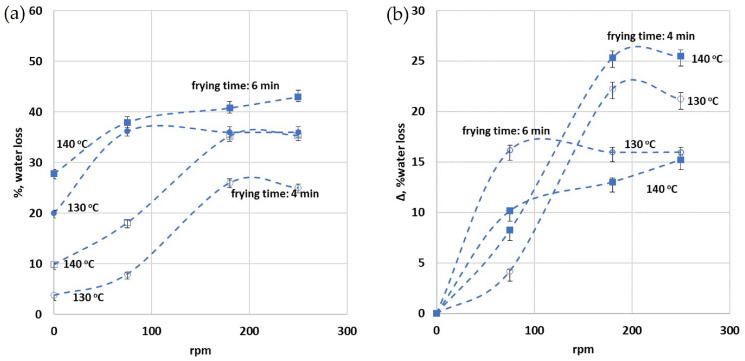
The figure illustrates the influence of varying rotational speeds (75, 180, 250 rpm) and frying durations (4 and 6 min) at an oil temperature of 130 °C and 140 °C on %, water loss. (**a**) %, water loss, (**b**) Δ, % water loss.

Figure 4a provides a thorough analysis of an experiment conducted to investigate the impact of various frying conditions on the percentage of water loss in food compared to the initial water content of the potatoes before the frying initiation (i.e., 80% initial water content corresponding to 44 g of water). The experiment was set up considering two initial oil temperatures (130 and 140 °C), two frying durations (i.e., 4 and 6 min), and a range of rotation speeds (from 0 to 250 rpm). The findings reveal that with the increase in rotation speed, frying duration, and temperature, there is a corresponding increase in the percentage of water loss. A positive correlation is established between the extent of water loss and the increase in rotational speed. This relationship was particularly noticeable at rotation speeds of up to 75 rpm for the 6 min frying duration, and up to 150 rpm for the 4 min duration. Notably, at the lower temperature of 130 °C, all rotation speeds failed to reach the critical 38% water loss, a threshold identified previously (Figure 3) as necessary for the food to be classified as palatable. The above results emphasize the importance of the rotational speed and frying temperature in effectively affecting water loss during the frying process.

Figure 4b compares the influence of the difference in the percentage of % water weight loss (Δ, % water loss) on various rotational field strengths (for two frying durations, i.e., 4 and 6 min) and two initial oil temperatures (130 and 140 °C). Δ, % water loss is calculated from the difference in % weight loss with and without the presence of the rotational field (the relevant data are presented in Figure 4a). The results show that for a frying duration of 4 min, an increase in the initial frying temperature to 140 °C consistently leads to a greater disparity in water loss between rotational and non-rotational frying conditions. However, for longer durations (6 min), the Δ, % water loss is higher for lower initial T_oil_ values. This trend can be explained in terms of the resulting water loss in frying at high initial oil temperatures and extended durations. In these conditions, one would anticipate an increase in water loss. Consequently, the Δ, % water loss—calculated based on the contrast between rotational and non-rotational frying conditions—would exhibit the observed pattern. This is due to the increased efficiency of the rotational field at enhancing water loss during frying at lower initial oil temperatures and shorter durations, where the water loss would obviously be less pronounced. This implies that there exists an optimal point between the rotational motion, the frying duration, and the initial oil temperature, where the benefits of using rotational frying are most noticeable.

Moreover, it is interesting to notice that Δ, % water loss stops increasing after 180 rpm for the 4 min frying duration (for both T_oil_ examined) and after 75 rpm for the 6 min duration for T_oil_ = 130 °C. For the 6 min frying duration at 140 °C, the Δ, % water loss increases by 10% at 75 rpm and then another 5% until 250 rpm. This indicates that the most profound effect of the rotational field is achieved upon reaching 75 rpm. The above observations clarify that the rotational field further enhances the frying process, especially at higher temperatures and a longer frying duration. Moreover, the most profound effect of the rotational field on frying is achieved during moderate rotational speeds.

**Figure 5:**
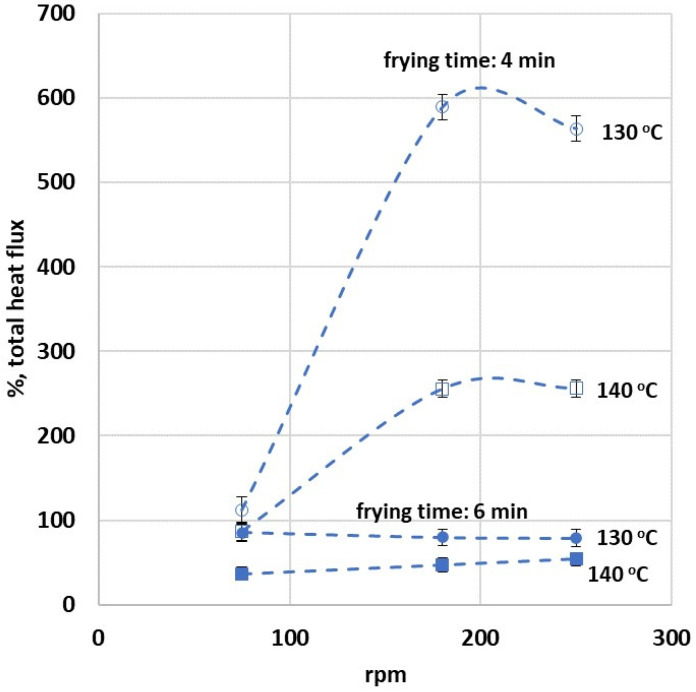
Impact of varying frying conditions—including initial oil temperature (130 and 140 °C), frying duration (4 and 6 min), and rotational speed (0 to 250 rpm) of the planetary fryer—on the percentage of the total heat flux during frying as calculated between rotational and non-rotational conditions.

Figure 5 presents the influence of the initial oil temperature (set at 130 and 140 °C), frying duration (spanning 4 and 6 min), and rotational speed (ranging from 0 to 250 rpm) on the percentage of the total heat flux. The percentage of total heat flux is indicative of the disparity in water loss observed under rotational and non-rotational conditions. Identical percentages of total heat flux should correspondingly mirror the same percentages in water loss between these two states, given that the total heat flux computation is fundamentally based on the quantification of water loss.

Figure 5 demonstrates that the application of a rotational field notably modifies the total percentage of heat flux. This suggests the amplification of the frying process within such a field. Nevertheless, the impact of the rotational field is considerably heightened during brief frying intervals (4 min) as opposed to more extended frying periods (6 min). This differential effect is due to the diminished water loss experienced during shorter frying durations, culminating in a sharper relative difference in total heat fluxes. This can be attributed to the lower water loss incurred during shorter frying times, which in turn leads to a more pronounced percentage difference in total heat fluxes. Regardless, for longer frying durations, the percentage water loss and corresponding heat flux are observed to be 47% and 80% higher, respectively, in the case of rotational frying compared to non-rotational frying (for 140 and 130 °C, respectively). This disparity is even more striking when examined in terms of heat flux, with calculations showing increases of 250% and 558%, respectively (for 140 and 130 °C, respectively). The fact that the % total heat flux is smaller for the higher T_oil_ could be attributed to the lower water loss incurred during lower oil temperatures. These findings highlight the significant influence of the rotational field on both water loss and heat flux during the frying process.

### 3.3. Effect of Initial Oil Temperature on % Water Weight Loss for Constant Oil Temperature

**Figure 6:**
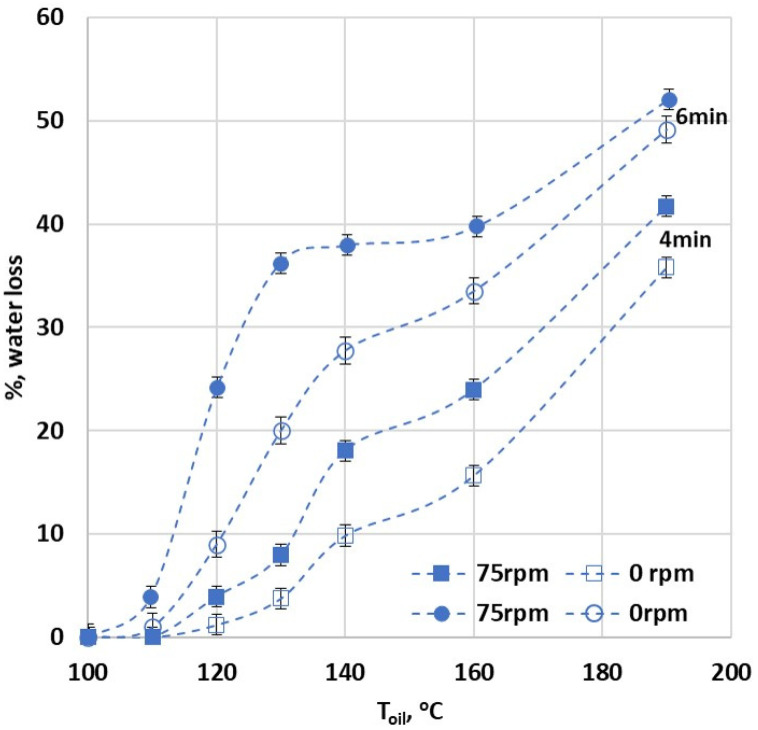
Impact of varying frying conditions—including initial oil temperature (130 and 140 °C), frying duration (4 and 6 min), and rotational speed (0 to 250 rpm) of the planetary fryer—on the percentage of %, water loss.

This section examines the pattern of water loss percentage during frying under a range of initial oil temperatures (from 110 to 190 °C). It considers typical mass-to-oil frying conditions with and without rotation at 75 rpm, a speed previously established as having the most substantial effect on water loss. Figure 6 presents the percentage of water loss (vertical axis) as a function of oil temperature (horizontal axis) for two frying durations (4 and 6 min), denoted by squares and circles, respectively. Unfilled symbols represent conventional deep frying, while filled symbols indicate 75 rpm rotation during frying. The red line marks the 38% water loss threshold, below which the fried product is considered inedible (as shown by the sensory assessment presented in Figure 3).

The data in Figure 6 demonstrate that, as also shown so far, moisture loss increases with prolonged frying times and increased oil temperature. Importantly, frying under rotation results in a significant rise in moisture loss at each initial oil temperature examined. For instance, at a frying time of 4 min and 190 °C without rotation, the fried product exhibits water loss slightly above 35%. In contrast, under rotation, water loss surpasses 38%. Moreover, for a frying duration of 6 min without rotation, water loss exceeds 38% only at 190 °C. However, with rotation, water loss is close to 38% even at 130 °C. The above results show that with rotation, a nearly 38% moisture loss can be achieved at a significantly lower temperature of 130 °C with a longer 6 min duration. Without rotation, this level of moisture loss is only reached at the much higher temperature of 190 °C. This suggests that rotation not only enhances moisture loss, but can also achieve comparable results at lower temperatures, thus potentially improving energy efficiency in the frying process.

**Figure 7:**
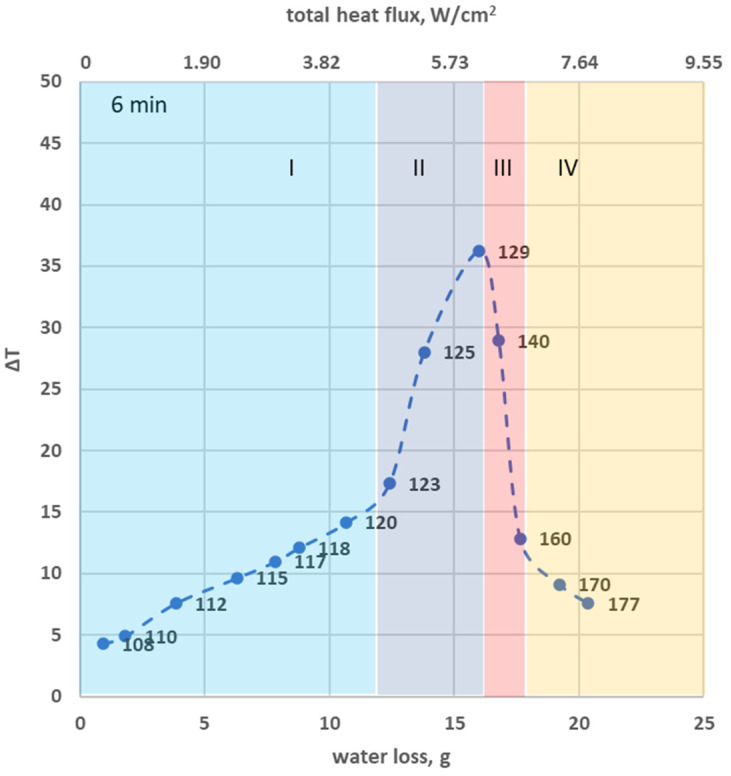

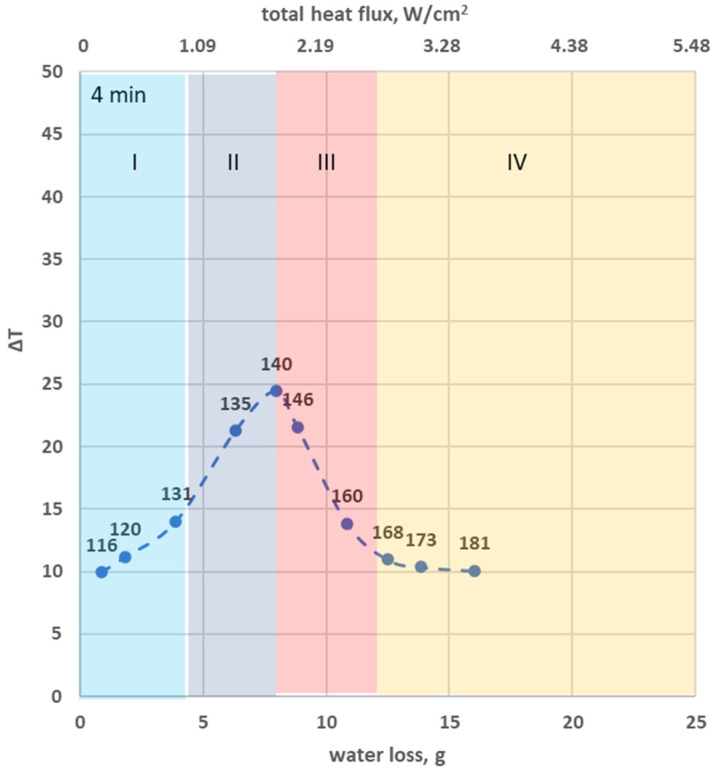
Relation between excess temperature (ΔT), water loss (g), and the total heat flux (W/cm^2^) during two examined frying durations. The temperature denoted at each point refers to the initial condition during rotational frying. The graph employs dual horizontal axes—the lower axis represents the water loss during frying, and the upper axis denotes the related heat flux. Each distinguished region is denoted by different colors and unique identifiers.

To explore how the excess temperature (ΔT)—defined as the temperature difference required in non-rotational frying to attain equivalent water loss as in rotational frying—correlates with the absolute water loss (measured in grams) and the corresponding heat flux due to water evaporation (measured in W/cm^2^), Figure 7 displays the relationship between water loss percentage (lower horizontal axis) and heat flux (upper horizontal axis) for various initial oil temperatures on ΔT for two observed frying durations (i.e., 4 and 6 min). To construct Figure 7, specific values were selected from the vertical axis of Figure 6, representing varying percentages of water loss. For these selected values, the corresponding initial oil temperatures under both rotational and non-rotational conditions were determined. These data were extrapolated from Figure 6, with temperatures estimated through interpolation among the initial oil temperatures denoted on the horizontal axis. Subsequently, the excess temperature (ΔT) was defined and calculated for each given percentage of water loss and initial oil temperature, all of which are presented in Figure 7. (The percentage of water loss can be easily transformed into absolute water loss values since the initial water content of the potatoes is known).

From Figure 7, it becomes apparent that four different regions can be recognized concerning the dependence of ΔT with %, water loss:

- Region I: A linear increase in ΔT with increasing % water loss is evident up to certain initial oil temperatures; 131 °C for a 4 min frying duration and 120 °C for a 6 min duration, linked to water loss percentages of 9% and 24%, respectively. This pattern demonstrates the heightened efficiency of a rotational field in promoting evaporation, resulting in similar water loss even at reduced initial oil temperatures.
- Region II: Subsequently, as the initial oil temperatures rise, an expected increase in water loss percentage is observed. Concurrently, the temperature difference, ΔT, escalates at a more rapid pace, peaking at 140 °C for a frying duration of 4 min and at 129 °C for a 6 min duration. These peaks correspond to water losses of 18% and 38%, respectively. This apex denotes the greatest ΔT value, denoting the maximum difference in temperature required for non-rotational frying to achieve identical water loss, thus underlining the optimized benefits of the rotational field at these conditions.
- Region III: After these peak points, ΔT displays an inverse relationship with increasing water loss, indicating a decreasing rate; the rate of decline is more profound for a 6 min frying duration. This shows that there is a threshold for the rotational field effect on the frying process depending on the initial oil temperature and the total frying duration.
- Region IV: ΔT continues decreasing with % water loss but with smaller rates compared to Region III.

The prevailing heat and mass transfer phenomena observed in this study can be associated with the evaporation of water during the frying process. These phenomena, and the resulting peaks observed in the excess temperature (ΔT), are influenced by the rotational field, the duration of frying, and the initial oil temperatures. The above-observed behavior could appear because, beyond this point, further increases in oil temperature might not necessarily improve heat and mass transfer efficiency, especially if the food water content has been significantly reduced already. After this peak point, the ΔT decreases with increasing water loss, suggesting that the advantage of the rotational field becomes less significant as the frying process continues, possibly due to diminishing water content in the food.

## 4. Discussion

The present study offers insights into a novel type of frying process, illuminating how rotational speed, frying duration, and oil temperature significantly impact the culinary quality of fried potato products. Critical to this understanding is the role of water loss during frying, a key determinant of the edible status of the potato product. The influence of rotational speed on water loss during the frying process is found to be significant, particularly at higher initial oil temperatures. It is observed that increasing the rotational speed up to 75 rpm leads to a substantial increase in the percentage of water loss. Exceeding this specific rotational speed, any further amplification in the speed does not contribute to a significant augmentation in water loss. The minimal degree of this increase after this point denotes a potential threshold in the efficacy of rotational speed as a determinant of water loss during the frying process.

Moreover, low initial oil temperatures such as 130 °C fail to achieve the critical 38% water loss threshold, irrespective of the rotational speed. Further analysis of the effect of the initial oil temperature on water loss reveals that an increase to 140 °C leads to a more pronounced disparity in water loss between rotational and non-rotational frying conditions. This observation shows that lower frying temperatures in the presence of a rotational field can achieve optimal water loss and product palatability. The concept of excess temperature (ΔT), or the additional temperature required in non-rotational frying to replicate the water loss achieved in rotational frying for a specific duration of frying, presents a fascinating trend. The initial results depict a linear increase in ΔT with water loss for the lower initial oil temperatures, demonstrating the superior efficiency of a rotational field in promoting evaporation and similar water loss at reduced oil temperatures. However, as the initial oil temperature increases and higher % water loss values are expected, ΔT exhibits a steeper ascent, peaks at certain temperatures, and then intriguingly starts to decline. This shows that there is a threshold for the rotational field effect on the frying process depending on the initial oil temperature and the total frying duration.

## 5. Conclusions

This study underlines the crucial role of adjusting frying parameters such as the rotational speed, frying duration, and initial oil temperature to optimize water loss and thereby augment the culinary quality of fried potato products. By employing an innovative device that manipulates the acceleration values within a rotating fryer through planetary motion, the present work showcases the enhanced efficacy of the frying process. Specifically, by modifying the rotational speed, the same water content can be achieved even at significantly lower initial oil temperatures across a range of frying durations. Fundamentally, the process of frying necessitates substantial heat fluxes for the evaporation of water. Nonetheless, in the context of a planetary rotational field, this transformation of water to vapor can be actualized at significant lower oil temperatures, thereby augmenting the thermodynamic efficiency of the frying process. This significant breakthrough promises not only healthier fried food but also a more cost-effective production method, potentially redefining industry standards in food processing.

The successful performance of the fryer with EVOO suggests that it can handle oils with more favorable frying properties, such as higher smoke points and lower viscosities. Consequently, the findings of this study are likely relevant to a wide range of frying oils. While further testing on parameters such as oil absorption rates, textural analysis, colorimetric analysis, oil degradation, and flavor profile is necessary, this initial work highlights the potential of the new fryer to produce healthier food products in a more energy efficient way by frying in lower oil temperatures. This research lays the groundwork for further exploration of its functionality, with future studies expected to provide a more comprehensive evaluation of the fryer’s capabilities.

## 6. Patents

Patent App. No: 20170100130/30.03.2017 Hellenic Industrial Property Organization.

## Data Availability

The original contributions presented in the study are included in the article/Supplementary File , further inquiries can be directed to the corresponding author.

## Supplementary Materials

The following supporting information can be downloaded at: https://www.mdpi.com/article/10.3390/foods13121896/s1, Supplementary File S1: Videos of a planetary fryer, Supplementary File S2: Trajectories calculations in a planetary fryer [29].
