# Supplementary material for: Enhanced Frying Efficiency at Low Temperatures Utilizing a Novel Planetary Fryer
Source: Foods. 2024 Jun 17;13(12):1896. doi: 10.3390/foods13121896 (PMC11202604; doi:10.3390/foods13121896)
Supplement: Supplementary file 1 [file foods-13-01896-s001.zip › Supplementary file S2.pdf]

Supplementary File S2: Trajectories calculations in a planetary fryer

The combination of the two rotational motions creates a sophisticated motion pattern. In a two-dimensional system, the evolution of the position of a single point M located at the periphery of a fryer basket is defined by the following equation [29]:

$$\mathbf{OM} = [R\cos(\theta) + r\cos(\phi)]\mathbf{i} + [R\sin(\theta) + r\sin(\phi)]\mathbf{j} \qquad \text{(s2.1)}$$

where **i** and **j** are the unit vectors along the x and y axes respectively, **OM** is the position vector of point M, R is the distance from the center of the frying device to the center of the frying basket, r is the frying basket radius, θ, ϕ are the angular positions with respect to the central axis and frying basket axis respectively. The absolute acceleration, **a** can be calculated from:

$$\mathbf{a} = [-R\omega^2\cos(\theta) - rw^2\cos(\phi)]\,\mathbf{i} + [-R\omega^2\sin(\theta) + rw^2\sin(\phi)]\,\mathbf{j} \qquad \text{(s22)}$$

where ω, w are the angular velocities around central axis and frying basket axis respectively ($\theta=\omega t$, $\phi=wt$ where t is the time).

Figure **S1.*a*** shows the complex trajectory of point M, which is computed using Equation (s2.1). It is important to note that all calculations were carried out under two distinct conditions: counter-rotation (depicted in the left column) and co-rotation, allowing for a comprehensive understanding of the system behavior under these different rotational states. ***Figure*** **S1.*b*** presents the correlation between the angular velocity (ω) of the frying basket and α/g_{earth} where α is the magnitude of the horizontal acceleration vector. The plot delineates an increasing trend in the acceleration values, which range from 5 to 25 α/g_{earth}, as the rotational speed escalates. Moreover, ***Figure*** **S1.*b*** provides a comparison between the two rotational conditions, namely counter-rotation and co-rotation. In the co-rotation scenario, both the fryers and the central rotational axis rotational directions have the same direction. Conversely, in the counter-rotation condition, these two rotations occur in opposing directions. It is distinctly observable that the co-rotation condition presents higher fluctuations (at the same range of ω values) as compared to the counter-rotation condition. This increased variation under co-rotation underpins our decision to conduct the experiments primarily under the co-rotation condition.

(a) Counter-rotation | co-rotation

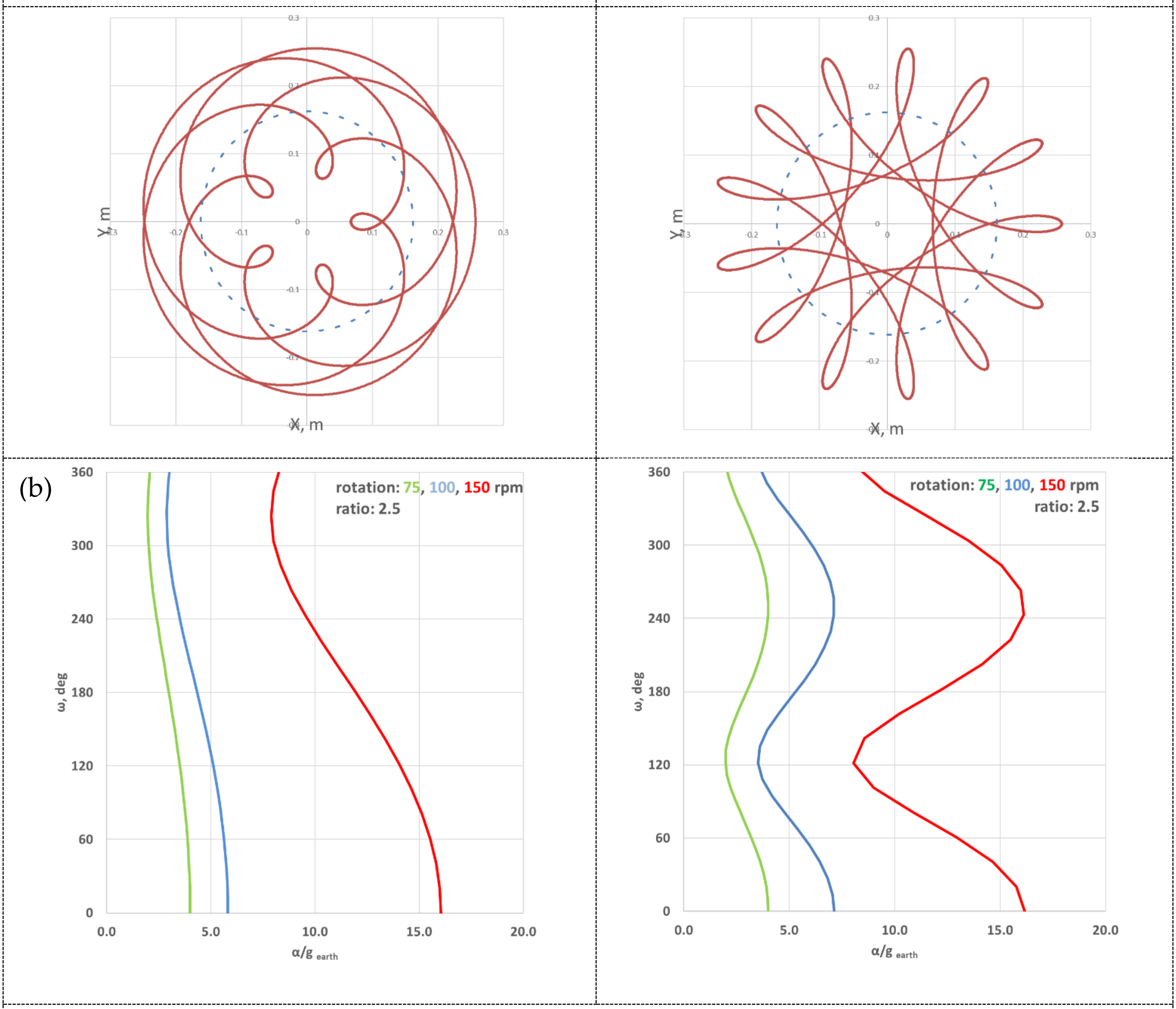

Figure S1 (a): first row; Depiction of a particle's complex trajectory as calculated from equation (2.3) (b): second row; Illustration of the impact of the frying basket angular velocity around central axis ω on the non-dimensional acceleration α/g_{earth} as rotational speed augments from 75 to 150 rpm. All calculations have been done both for counter-rotation (left column) and co-rotation conditions
